# The Impact of Rational Warm-Up on Physical Preparation and Injury Prevention in Young Footballers: A Longitudinal Study

**DOI:** 10.3390/jcm15020608

**Published:** 2026-01-12

**Authors:** Henryk Duda, Łukasz Rydzik, Tadeusz Ambroży, Pavel Ruzbarsky, Andrzej Kędra, Wojciech Wąsacz

**Affiliations:** 1 Department of Sports and Recreational Games, Institute of Sports Sciences, University of Physical Culture, 31-571 Kraków, Poland; henryk.duda@awf.krakow.pl; 2 Department of Sport Theory and Motor Skills, Institute of Sports Sciences, University of Physical Culture, 31-571 Kraków, Poland; tadek@ambrozy.pl (T.A.); wojciech.wasacz@awf.krakow.pl (W.W.); 3 Department of Sport Kinanthropology, Faculty of Sports, Universtiy of Presov, 080 01 Prešov, Slovakia; pavel.ruzbarski@unipo.sk; 4 Department of Sport Science, Faculty of Social Sciences, Vincent Pol University, 20-853 Lublin, Poland; aakedra@onet.pl

**Keywords:** football, physical preparation for exertion, post-exercise recovery, flagship team sport

## Abstract

**Background/Objectives**: One of the pillars of optimal footballer performance is the gradual preparation of the body for physical exertion in terms of intensity. The aim of this study was to evaluate the impact of a structured warm-up and cool-down program on flexibility, perceived fatigue, and injury prevention in young football players. **Methods**: Participants were 60 junior football players (U17), with a mean age of 16.5 ± 0.5 years, mean height of 172.5 ± 6.7 cm, and mean body mass of 70.2 ± 6.4 kg. The participants were assigned to experimental (EXP; n = 30) and control (CON; n = 30) groups during 8 mesocycles. A 4-week training stimulus was applied in parallel, consisting of an author-designed exercise routine with a profiled intensity (warm-up and cool-down parts) for the EXP group and standard exercises for the CON group. Selected variables (motor, endurance, injuries) were assessed before, during, and after the intervention. Additionally, the profile of selected correlations was analysed. Statistical analysis was performed using *t*-tests with a significance level set at *p* < 0.05. **Results**: In the EXP group (post-test), a significant improvement in flexibility was observed in the forward trunk flexion test (d = 1.13 cm; *p* < 0.001; d_c2_ = 1.05). Simultaneously, participants reported lower levels of subjective fatigue (RPE = 6.86 ± 0.82 points) compared to the CON group (*p* = 0.016; d_c_ = 0.46) and demonstrated fewer injuries during the annual cycle (0.97 ± 0.83 vs. 1.33 ± 0.66; *p* = 0.026; d_c_ = 0.48). Both groups showed a strong negative correlation between flexibility and the number of injuries in the annual cycle, training experience and the number of injuries, as well as training experience and RPE (all r_p_ > −0.50). A strong positive correlation was found between RPE and the number of injuries (r_p_ > 0.60). **Conclusions**: The results demonstrate that the structured warm-up and cool-down program significantly improved flexibility, reduced perceived fatigue, and decreased injury occurrence in the participants.

## 1. Introduction

Years of competitive and coaching experience, as well as scientific research, identify that the foundation of optimal performance in footballers’ physical activity is the gradual and moderate (in terms of intensity) preparation of their body’s adaptive processes for exertion. This state is primarily achieved through the selection of rational general development exercises (such as exercises with balls in football training) applied during the warm-up [1].

Previous observations indicate that, typically, during the warm-up before training or a match, exercises with high dynamics (e.g., SMALL GAMES) are introduced in too forceful a manner. This not only exposes the player to injury (due to insufficient preparation of the movement apparatus) but, more importantly, limits the full metabolic adaptation to exertion. This can have both immediate health consequences (increased risk of injury) and subsequent effects (overtraining of systems) [2]. Furthermore, this approach makes little use of the exertional, psychomotor, and technical potential of the players’ bodies. Consequently, this leads to the players not being fully prepared for physical exertion [3]. Also, during the training session, the rest period plays an important role. When the activity level is low and the duration is short, this rest period should be deliberately included in the training session. In such cases, the player actively enhances the adaptation of the muscular and ligamentous system to exertion by performing stretching exercises. During a warm-up, the body undergoes a variety of neural and physiological adaptations, such as increased motor unit recruitment, enhanced muscle elasticity, and improved blood flow to active muscles. These changes help prepare the body for more intense activity and reduce the risk of injury. Additionally, compensatory and recovery exercises after intense effort, especially in the final part of the training session, play a significant pro-health role [2,4]. It is important to remember that, in the context of intense football training, a rational and health-oriented approach to preparing the player for physical exertion is crucial for achieving optimal sports results and maintaining physical and mental health [2,3,4].

The issues surrounding this problem are highly relevant in the training of football players, as the specificity of their training significantly impacts the circulatory system. High dynamic activities, without a gradual adaptation of the body to exertion, can lead to overexertion and, in the long-term training process, result in serious health complications (heart defects and nervous system disorders) [2]. Additionally, excessive training loads (especially without proper warm-up) can have irrational effects on the movement apparatus, leading not only to microinjuries but also, over time, to tendencies for muscle-ligament structure contractures, muscle stiffness, which ultimately results in reduced joint range of motion. Restricted joint mobility can lead to abnormal loading, causing disturbances in their functioning and, consequently, injuries [5]. In rational training for football players, it is also important to consider the lack of “BODY RELAXATION” after intense exercises, as the physiological reactions occurring after exertion are not natural metabolic mechanisms in the functioning of this process [6].

In football training (also for children and youth), there has been a trend in recent years to emphasize training intensity from the very beginning of the training session, based on the principle “TRAINING LIKE THE GAME” [7,8]. This approach may seem justified, as effective training should be based on the specificity of actions and effort characteristic of the actual game. However, the preparation of a football player for physical exertion should take into account the fact that physiological laws indicate the need for gradual engagement of the movement apparatus, oxygen supply mechanisms (circulatory and respiratory systems), and the psychological sphere (exercises from easy to difficult, from less intense to more intense). Such methodological [4] and physiological [2,3,6,9] principles not only facilitate learning actions but, above all, have a pro-health dimension and enable long-term sports success. A footballer’s preparation for physical exertion should take into account physiological principles, which emphasize the need for gradual engagement of the musculoskeletal system, the oxygen-supplying systems (circulatory and respiratory), and psychological aspects. This involves progressing from simpler to more complex exercises and from lower to higher intensities, in accordance with methodological [4] and physiological [1,2] principles, which not only facilitate skill acquisition but, above all, have health-promoting value and enable long-term sporting success.

The above observations regarding the training content in constructing a training session seem concerning, as the current training trend in football players, associated with high intensity, already applies to the initial part, the so-called “WARM-UP”, where very dynamic forms of games, including individual duels, are often introduced without preparing the body for exertion. Also, in the final part, instead of relaxing (“CALMING”) exercises, exercises aimed at correcting training deficits are introduced in the form of game fragments and game play (even extending the actual game), which are characterized by high intensity (similar to the main part of the session), followed by a full cessation of movement activities at the end of the training [7]. Therefore, the question arises: Does the construction of a training session based on such principles have a health-promoting character for football players?

Considering the outlined criteria, a research process was decided upon, focusing on a group of young football players in the junior category. The aim of this study was to assess the impact of a general developmental exercise program with an emphasis on mobilization, featuring increasing intensity (warm-up) and gradually decreasing intensity (cool-down), on the mobility of the musculoskeletal system and functional preparation for physical exertion in young football players.

Based on a review of the literature and extensive experience in coaching and playing, we hypothesized that the application of general developmental exercises with adjusted intensity, incorporated into the football warm-up and cool-down parts, would significantly contribute to the optimization of functional preparation for physical exertion in young football players. The aim of this study was to evaluate the effects of a general development program emphasizing mobility, characterized by progressively increasing intensity during the warm-up and gradually decreasing intensity during the cool-down, on musculoskeletal mobility and functional readiness for physical exertion among young football players.

## 2. Materials and Methods

This study was a multi-stage longitudinal trial conducted at The University of Physical Education in Krakow and the Faculty of Sport at the University of Prešov, focusing on young football players. The project was approved by the Bioethics Committee of the District Chamber of Physicians in Kraków (No. 97/KBL/2015). In accordance with the requirements of the Helsinki Declaration, participants and their legal guardians were informed about the research objectives, methods, potential side effects, and their right to withdraw from the study at any time without providing a reason. Written informed consent was obtained from all parents and legal guardians for participation in this study.

### 2.1. Study Design

The evaluation of training effectiveness was conducted using the pedagogical experiment method. The parallel, rotational group technique was used: experimental and control groups [10]. The multi-stage longitudinal study was conducted as an experiment over 8 mesocycles (1 mesocycle—a 4-week training plan), annually over a period of 8 years (2015–2019 and 2022–2024), totaling 8 months of training. Additionally, in each of the mesocycles, an independent group of U17 players (rotational groups) was selected based on uniform inclusion criteria (age, skill level, discipline). The selection for the experimental group (EXP) and control group (CON) was done separately in each mesocycle, adhering to the age range of the participants (U17—age from 16 to completion of 17 years). The U17 age category was selected because it represents a crucial developmental period for young athletes, particularly in football. At this age, players undergo significant physical and psychological changes that affect their ability to achieve high performance. Training during this period is key to establishing long-term athletic development, and properly planned warm-up and cool-down routines may provide substantial benefits in terms of injury prevention and performance optimization. This allowed for averaging results between groups and assessing the overall effect of the intervention in the athlete population, as presented in the report. The testing procedure was carried out before and after each 4-week experimental intervention period. The level of flexibility, subjective assessment of exercise intensity (RPE), and the injury profile of the lower limbs in the annual training cycle were verified. In the EXP group, the intervention was integrated into their regular training program, supplemented with special exercises in the initial and final parts of the training session (targeted heart rate frequency and exercise content). The CON group followed their standard training program. The experimental stimulus was applied to reliably compare the training effects obtained with the rational warm-up conditions against the effects of traditional methods commonly used in sports clubs.

### 2.2. Participant Characteristics

In the 8 training mesocycles, a total of 60 junior football players aged 17 (U17) from Poland and Slovakia, training professionally, were selected for the study through purposive sampling. The sample size was a priori estimated using G*Power v.3.1.9.6 for the *t*-test (2 independent groups, comparing pre- and post-intervention results). A medium effect size was assumed (Cohen’s f = 0.25; equivalent to d = 0.5), with a significance level of α = 0.05 and a target power of the test (1 − β) = 0.80. The calculations showed that a minimum of 34 participants (17 in each group) was required to achieve statistically significant differences. Ultimately, 60 participants (30 in the EXP and CON groups) were tested, which exceeded the a priori requirements and ensured adequate statistical power for conducting comparative analyses. For dependent samples, the analysis showed that detecting significant differences before and after the intervention required a minimum of 27 participants, indicating that the sample was sufficient to meet the requirements and to perform comparative analyses in the context of the research project. Participants were randomly assigned to the experimental or control group using a computer-generated randomization schedule.

A total of 128 participants were initially recruited (8 participants in each 4-week training period for both the EXP and CON groups). These participants were football players from the School of Sports Mastery in Krakow (Poland), randomly selected clubs from the Małopolska and Subcarpathian regions (Poland), as well as juniors from the Prešov region (Slovakia). During the experimental intervention, 68 of them were excluded from the study due to acquired injuries, current health conditions, or voluntary withdrawal without providing a reason (n = 26). Additionally, some results were discarded due to the minimum attendance requirement (n = 42). In the final analysis, only 60 participants who met the minimum attendance requirement of 90% were included. Inclusion criteria for the study included: a minimum of 5 years of training experience, current medical clearance, no history of severe injuries, a positive medical recommendation, no use of supplements (during the study period) or doping, and active participation in competitions. Exclusion criteria included any conflict with the above aspects, current injuries or conditions that might affect participation, and lack of parental consent for participation in the study. The mean body mass of the participants was 70.30 ± 6.20 kg, with an average height of 172.08 ± 6.82 cm. The mean BMI index was 23.62 ± 1.84. The age of the participants ranged from 16 to 17 years (mean age: 16.56 ± 0.51 years). Training experience ranged from 5 to 10 years of systematic training, with 4 to 5 training sessions per week, depending on the training cycle (mean experience: 7.57 ± 1.22 years). The study was conducted during the preparatory period. Information on the participants’ age, training experience, activity, and competition history was obtained through a diagnostic survey using direct interviews with the juniors and coaching staff.

Group selection procedure (methodology): in each of the mesocycles, 16 participants (U17) were recruited, with 8 being intentionally assigned to the experimental group (EXP; n = 8) and 8 to the control group (CON; n = 8). As mentioned previously, the results from 8 mesocycles over 8 years with rotational groups were averaged for participants who met the inclusion criteria (n = 60). Group selection was based on an organized selection method [10], where players were classified using a ranking number table. This procedure eliminated randomness, generating rotational groups with similar training and sports levels, age, and basic structural profiles, without significant differences (all *p* > 0.05; Table 1).

### 2.3. Characteristics of the Experimental Intervention

The intervention involved a 4-week training application (mesocycle). Both the EXP and CON groups participated in the planned training sessions four times a week (90 min each) throughout the entire period, with the same subject matter for each session (teaching and improvement goals). The content, themes, and intensity of the main part of the training session (50–60 min) were identical for both groups, ensuring comparable loading conditions and work scope. The only difference in task content between the groups was in the initial and final parts of each training session.

In the EXP group, the initial part (20–25 min) involved exercises with balls, directly related to the session’s theme. These exercises were carried out in strict form and in general developmental game fragments. The intensity of effort in this part gradually increased from low to moderate, corresponding to a heart rate range of HR = 100–140 beats per minute (bpm). To develop and improve flexibility [11] and activate muscles, these exercises were alternated with dynamic stretching. In the final part (10–15 min), the EXP group performed exercises of medium and low intensity, aimed at addressing training deficits and gradually reducing the level of physical activation. Relaxation and calming exercises were applied, interspersed with static stretching at a moderate heart rate of HR = 120–140 bpm. A unique feature was the profile of general developmental exercises aimed at the main task, flexibility exercises, and the intensity level.

On the other hand, the CON group followed a standard warm-up and cool-down program according to the general cycle, typical for club practices. In the initial part of each training session (20–25 min), exercises with balls were performed in line with the session’s theme, with a dynamic nature. The exercises were mostly carried out in game fragments and task-oriented games, often in 1 × 1 configurations, promoting intense motor and energy engagement from the players. This part of the session was of high intensity, with moderate or near-high heart rate frequencies at HR = 130–160 beats per minute (bpm) [12]. In contrast to the EXP group, no stretching elements or exercises with reduced intensity were included. The warm-up had a fully dynamic and task-oriented nature. In the final part of the training session (10–15 min), high-intensity exercises were used, aiming to address the training deficit and maintain a high level of effort. No calming or static stretching exercises were introduced. The average heart rate in this part of the session was HR = 140–160 bpm, confirming the maintenance of intense physiological loading throughout the entire final phase of the training session [12].

In total, during the 8 interventions (over 8 years), 104 training sessions were conducted (9360 min, including missed sessions). The number of sessions was the same for both groups. The training time for one intervention (a 4-week mesocycle) was 1170 min, with approximately 293 min per week. The training interventions in both groups were led by experienced, certified coaches who were trained by the project authors. Efforts were made to ensure that the same coaches led the sessions throughout the study. Instruction was provided regarding suggested meals to maintain a balanced diet, appropriate rest, and consistent training intensity (following the coaches’ recommendations, with attention to punctuality and presence). Attendance was recorded in a logbook.

**Figure 1:**
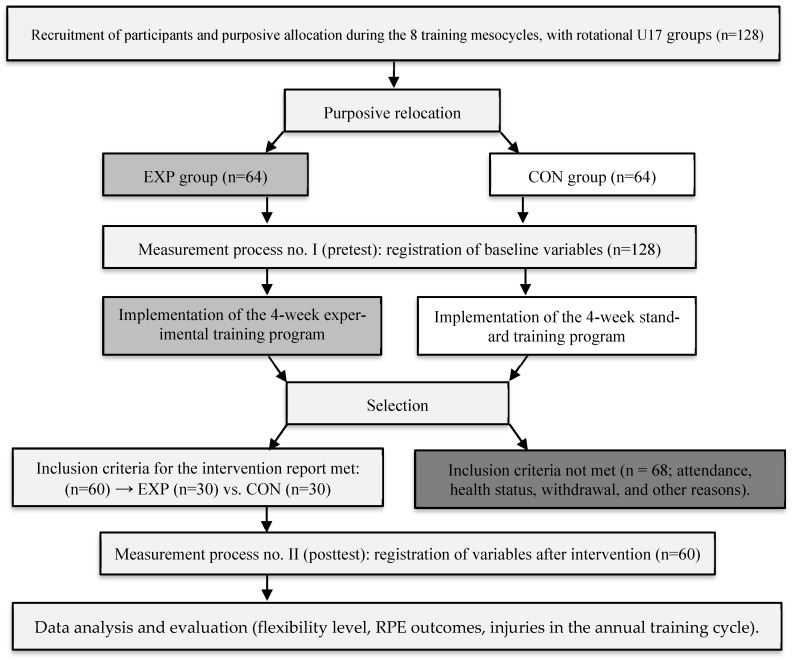
Flowchart of the research intervention.

To determine the profile of changes induced by the application of the training stimulus, two measurement points were conducted in each mesocycle. The first was before the start of each of the 8 mesocycles (baseline assessment of variables—pretest). The second measurements were taken after the completion of the 4-week training period (evaluation of effectiveness—posttest). The impact was assessed in relation to the results obtained within the EXP group (within-group) and compared with the CON group (between-group), which served as the reference point for comparison. Figure 1 presents the block diagram of the research intervention.

### 2.4. Measurement Procedure

- Assessment of Flexibility and Motor Abilities

Before each measurement session, both groups participated in a 15-min warm-up session consisting of exercises to prepare the body for physical activity. A series of shaping exercises was used, including static and dynamic movements of the arms, torso, abdomen, back, legs, and head. The examiner then demonstrated each test according to the procedure and provided instructions and explanations. The measurements took place at specially prepared stations (gymnasium), with only one group present during each measurement session. Participants were instructed to refrain from physical activity for 24 h prior to the testing session. All tests were conducted at the same time of day (between 4:00 PM and 6:00 PM) to minimize the effect of diurnal fluctuations. Effective recovery breaks (minimum 20 min) were provided between tests. The testing procedure included the following trials:

1. Forward Trunk Flexion in Standing Position [13]. Assessment: flexibility level of the posterior chain muscles, mainly the hamstrings and the lumbar spine. Procedure: the participant stood barefoot on a specially prepared measuring platform (height 20 cm) with feet together. The participant extended their arms forward and slowly bent their torso forward, trying to reach as low as possible without bending their knees and without jerking movements. The hands moved along a centimeter-scale ruler placed in front of the platform. The measurement was taken twice, and the better result was used for analysis. The value was recorded in cm as the lowest point the fingers could reach. A negative result meant the participant could not reach the level of the feet (below “0”), while a positive result meant the participant exceeded the feet level (above “0”). Equipment: platform with an accurate centimeter scale (ranging from −20 cm to +20 cm), research sheet. Control recommendations: straight knees, slow and controlled movement without jerks or forced actions. Interpretation: results were analyzed in raw form (cm).
2. Standing Long Jump [14]. Assessment: explosive strength. Procedure: the participant stood with feet slightly apart behind the starting line, bent their knees, swung their arms backward, and then jumped forward (also moving their arms forward) as far as possible, landing on both feet while maintaining a standing position after landing. The test was performed twice, and the best result was recorded. The distance was measured to the nearest heel mark with an accuracy of 1 cm. Equipment: measuring tape, two connected gymnastics mats, research sheet.
3. 10 × 5 m Shuttle Run [14]. Assessment: muscle mobilization speed, running endurance, and agility hybrid. Upon signal, the participant sprinted to the second line 5 m away, crossed it with both feet, and returned. This sequence was repeated 10 times. The shuttle run time was recorded and rounded to the nearest tenth of a second. Equipment: stopwatch, markers, chalk, research sheet.
4. Flamingo Balance Test [14]. Assessment: static balance. The participant stood on one foot on a balance beam (50 cm long, 4 cm high, and 3 cm wide) while holding the opposite foot with a bent knee. The goal was to maintain balance for as long as possible. The measurement ended when the participant lost balance, released the foot, or touched the ground. A preparatory trial was allowed before the measurement. Time was measured with an accuracy of 0.01 s. The test was conducted according to Żak’s modification [15]. Equipment: balance beam (Benz Sport, Winnenden, Germany), stopwatch (Casio, Tokyo, Japan), research sheet.

B. Subjective Perception of Exercise Intensity (RPE) Evaluation

The subjective perception of effort (RPE) was assessed using the CR-10 scale [16]. One minute after each training session, participants answered a question regarding their perceived effort on a scale from 0 to 10, where 0 represented no effort, 5 represented moderate effort with the ability to continue comfortably, and 10 indicated maximum effort requiring immediate cessation and rest. All participants were instructed and familiarized with the scale before the test. The results from the 4-week intervention were averaged and presented in the report.

C. Diagnosis of Lower Limb and Trunk Injuries—Documentation Analysis

To identify and classify injuries to the lower limbs and trunk among the participants, a retrospective analysis of medical and training documentation was conducted, covering the entire annual training cycle (post-intervention). Data sources included individual medical records and training documentation (notes and training logs with comments on pain, range of motion limitations, training absences, and load adaptations). The analysis included all reported complaints, injuries, or traumas to the lower limbs and trunk that occurred during training, competitions, or recovery periods. The cases were quantitatively classified without specifying the exact location within the musculoskeletal system.

### 2.5. Statistical Analysis

Normality of data was assessed using the Shapiro–Wilk test. Parametric data were analyzed using *t*-tests, while non-parametric data were analyzed using Mann–Whitney U tests. Homogeneity of variance was tested using Levene’s test, and if violated, Welch’s test was applied. Effect sizes were calculated using Cohen’s d (d = 0.20 for a small effect, d = 0.50 for a moderate effect, and d = 0.80 for a large effect). To assess the relationship between the variables, Pearson’s linear correlation was used. The correlation thresholds were as follows: rp = 0.0 to 0.19 for very weak correlation, rp = 0.20 to 0.29 for weak correlation, rp = 0.30 to 0.49 for moderate correlation, rp = 0.50 to 0.79 for strong correlation, and rp > 0.80 for very strong correlation. The collected data were analyzed using Statistica software, version 13.3 (Statsoft, Kraków, Poland). The statistical significance level was set at *p* < 0.053.

## 3. Results

**Figure 2:**
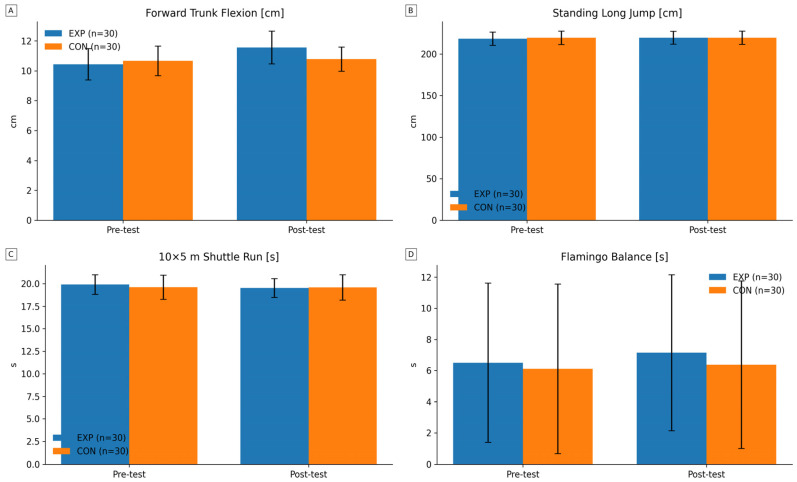
Changes in physical fitness and mobility outcomes in the experimental (EXP, n = 30) and control (CON, n = 30) groups before (pre-test) and after (post-test) the intervention. Bars represent mean values, and error bars indicate standard deviations (SD). (**A**) Forward trunk flexion in standing position [cm]. (**B**) Standing long jump [cm]. (**C**) 10 × 5 m shuttle run [s]. (**D**) Flamingo balance [s].

Table 2 and Figure 2 present the comparative results of the flexibility test and motor performance tests in the examined groups of youth football players (EXP vs. CON).

In the baseline intergroup assessment (pre-test), the experimental and control groups (EXP vs. CON) demonstrated a comparable profile of flexibility and motor performance variables (all *p* > 0.05).

Following the intervention period (post-test), a significant improvement in flexibility was observed in the EXP group, while no significant changes were noted in the CON group. The intergroup analysis showed that, after the intervention, the EXP group achieved a significantly higher score compared with the CON group, confirming the effectiveness of the applied program in developing flexibility. The low coefficient of variation values (CV 9–10%) indicate high measurement repeatability and homogeneity of the tested sample.

The statistical analysis did not reveal any significant differences between the EXP and CON groups in any of the motor test outcomes. The EXP group achieved more favorable training effects (long jump d = 1.27 cm; 10 × 5 m shuttle run d = −0.4 s; balance test d = 0.65 s), although these differences did not reach statistical significance. The coefficient of variation values indicate high repeatability of results in the long jump and shuttle run tests (CV 3–7%), with greater variability observed in the balance test (CV 70–90%).

The comparative analysis revealed significant between-group differences in the level of subjective perceived exertion and in the number of lower-limb and trunk injuries recorded over the annual training cycle. Players in the EXP group reported lower RPE values compared with the CON group, which may indicate greater energetic efficiency and improved adaptation to physical effort. Simultaneously, this group showed a retrospectively lower number of injuries than the CON group. The obtained effect size values (d ≈ 0.5) indicate a moderate but practically meaningful impact of the applied intervention (Table 3).

Correlation analysis revealed significant associations between selected variables in both study groups. A strong negative correlation was observed between flexibility (lower muscular tightness) and the number of lower-limb injuries, suggesting that a higher level of flexibility may reduce the risk of injury. A strong positive correlation was found between RPE and injury incidence, indicating that players reporting higher perceived fatigue experienced more injuries during the annual training cycle. In both groups, significant negative associations were noted between training experience and the number of injuries, which may suggest a protective effect of training seniority. Additionally, a negative correlation was identified between training experience and RPE, indicating that players with longer training experience perceived the same exercise load as less intense (Table 4).

## 4. Discussion

The results of this study provide valuable insights into the role of structured warm-up and cool-down routines in preparing young football players for physical exertion. Particularly important was the effect of a progressively increasing warm-up intensity on musculoskeletal mobility, as well as on perceived fatigue and the number of injuries over a one-year training cycle. The key findings of the present intervention demonstrated that the experimental training program—a combination of general developmental and mobilization exercises with profiled intensity—had a positive impact on flexibility, perceived fatigue, and injury prevention across the annual training cycle in U17 football players from the EXP group (Table 2 and Table 3). Following the intervention, participants exhibited a significantly higher level of posterior-chain flexibility, particularly in the hamstrings and lumbar region. Furthermore, based on immediate post-training feedback, players reported lower subjective fatigue compared with the CON group. Our findings are consistent with previous studies highlighting the importance of structured warm-up routines in improving both athletes’ physical and psychological performance. For example, the study by Rössler et al. showed that a progressive warm-up leads to improved sprint performance and a reduced number of injuries among young football players [16]. Importantly, retrospective analysis also revealed a reduced number of lower-limb and trunk injuries over the annual training cycle following the intervention. Another notable finding concerns the relationships between the applied training stimulus and the measured variables (Table 4). An inverse association was observed, showing that fewer injuries co-occurred with higher flexibility levels. In contrast, greater perceived fatigue (RPE) was associated with a higher frequency of injuries.

An optimal range of flexibility is a highly important training component, as it can significantly influence the functional efficiency of a football player’s musculoskeletal system [5]. The flexibility improvements observed in the present study confirm that the applied experimental procedure produced the intended effects, and the obtained results may serve as a model for promoting health-oriented training in football. Moreover, the demonstrated relationships suggest that developing and improving flexibility contributes to a lower incidence of training-related injuries.

Excessively intense, even short-duration exercise can disrupt the body’s adaptive response to training [17]. This results from insufficient activation of anaerobic pathways, which limits metabolic efficiency under rapidly increasing load [18]. At the molecular level, warm-up exercises enhance the activity of enzymes involved in energy production, improve the elasticity of muscle fibers, and optimize the function of the nervous system, all of which contribute to improved performance and injury prevention. The consequence is accumulating fatigue associated with oxygen deficit, leading to decreased motor performance during subsequent exercise and elevated subjective fatigue after its completion [17]. In the present study, this issue was examined by assessing the players’ perceived exertion (RPE) after each training session, as well as by retrospectively analysing injury incidence during the annual training cycle following the intervention. Players in the EXP group—who were exposed to gradual, low-to-moderate training loads in the warm-up phase, with emphasis on general-development and flexibility exercises—reported lower subjective fatigue and sustained fewer injuries compared with the CON group, which performed strenuous activities (small-sided games and full games) both in the warm-up and cool-down parts of the session. The observed relationships indicate that lower RPE values were associated with a reduced number of injuries. This confirms that effective preparation for intense exercise requires progressive physiological adaptation, whereas the end of the session (cool-down) should facilitate a gradual return to baseline. Such a strategy appears essential for rational training design [9]. This method of preparation reduces the risk of musculoskeletal injuries [19]. These findings are consistent with existing literature [5,19,20], which indicates that progressive, low-intensity general-development and stretching exercises optimize the athlete’s readiness for training, improve motor function, enhance exercise tolerance, reduce injury risk, and support long-term athletic development. The structured training programme implemented in the EXP group contributed to lower perceived exertion and fewer injuries, demonstrating its positive influence on physiological adaptation and its clear health-promoting value.

For the motor abilities assessed through the applied tests, comparative analyses did not reveal statistically significant changes. However, it is important to emphasise that the magnitude and consistency of the observed improvements varied. The training effects demonstrated a favourable direction of change in the EXP group (long jump: +1.27 cm; 10 × 5 m shuttle run: −0.4 s; balance: +0.65 s; Table 2). It is possible that achieving greater improvements in these variables would require longer exposure periods, which should be considered in future research. Interestingly, our findings make it possible to identify directional trends in the optimisation of motor performance in the EXP group compared with the control group. This suggests that the applied programme of rational warm-up and cool-down provides additional benefits manifested as improvements in selected motor variables. Such outcomes support comprehensive athletic development and are consistent with the findings of a recent systematic review, which indicated that fascial release and flexibility exercises may contribute to improvements in explosive strength and balance [21].

Interestingly, our findings also revealed additional significant relationships within the entire cohort of junior players (both groups). Training experience showed a strong negative association with the number of reported injuries, suggesting that more experienced athletes tend to sustain fewer injuries. Furthermore, a strong negative relationship was observed between RPE and training experience, indicating that more experienced players perceive the same physical effort as less intense. These observations highlight the need for broad individualisation in coaching practice. Athletes with lower training experience require more time for recovery between training sessions, in contrast to more experienced players who demonstrate greater adaptive capacity to training loads.

In summary, contemporary football training increasingly exhibits patterns that raise concern, as traditional practice often disregards the principle of gradually preparing the body for physical exertion [7]. Already in the warm-up phase, high-intensity, competitive activities are introduced, justified by the need to replicate game specificity and accelerate adaptation to match conditions [8]. Similarly, in the final part of the training session, instead of calming, cooldown-oriented exercises, coaches often introduce activities aimed at “compensating training deficits”, extending the session by continuing dynamic, high-intensity drills (sometimes even small-sided games) [7]. Such practices indicate a departure from health-oriented principles in football training. They may reduce training effectiveness and, more importantly, pose significant health risks—especially for young players. Considering the findings of our study, several practical recommendations emerge. In sports training—particularly in the warm-up phase—it is advisable to implement general developmental and dynamic stretching exercises, applied according to the principle of gradually increasing exercise intensity [2]. These activities can be combined with ball-related elements, provided they follow the methodological rule of progressing from simple to more complex forms. Such an approach ensures safe preparation for exertion and positively influences the health of young players [2,3]. Rational training that uses dynamic, high-intensity activities is essential in team sports; however, it must be preceded by a structured, gradual preparatory process. Due to the physiological nature of football, in which aerobic metabolism predominates, this preparation should begin early in the session as a physiological “activation phase” for the body [18,22]. General developmental exercises serve not only to activate the musculoskeletal system but also to provide compensatory and pro-health effects, supporting overall functional balance and performance [18,23]. In the final part of the training session—after high-intensity work—gentle cooldown exercises should be introduced, ideally using the ball to preserve football specificity. Such activities support the muscle pump, accelerate cardiovascular recovery, enhance oxidative processes, and reduce muscular acidosis [6]. As highlighted by Birch et al., these exercises aid in removing post-exercise metabolites, dissipating heat, lowering psychophysiological tension, and stabilising cardiovascular function, reducing the risk of arrhythmias [2]. Adding stretching further enhances muscle functionality and reduces the risk of injury [5,19]. These recommendations are particularly important for youth training. Young footballers often follow a demanding weekly schedule (up to five sessions plus competitions), and during developmental years—when the body is not yet fully mature—their muscles and joints are especially vulnerable to overload and injury [2]. Proper physiological preparation for exertion is therefore crucial for both performance and long-term health.

### Limitations of the Study

Despite careful design, organization, and implementation of the study, several limitations should be acknowledged, as they may affect the generalizability and interpretation of the findings. One limitation is the sample size. Although it was determined based on power analysis, increasing the sample size would improve statistical power and reduce the risk of Type II errors. Another limitation concerns the specific profile of the participants (U17 male football players), which may limit the applicability of the findings to other populations of athletes in team sports (e.g., basketball, volleyball, handball). Additionally, the sample consisted exclusively of male participants in the early stages of their athletic development, so gender-based comparisons were not possible. To capture a broader and more representative context, future research should include larger sample sizes, various age groups, and female cohorts. Finally, it is recommended that research efforts be expanded to include other team sport disciplines (TSD) to further investigate the applicability of the findings in different sports contexts.

## 5. Conclusions

Our findings demonstrate that a rational warm-up and cool-down program significantly improved flexibility, motor performance, subjective fatigue, and injury prevention among U17 football players. The EXP group achieved better post-test outcomes compared to the CON group, with improvements in flexibility and a positive trend in motor abilities, though not statistically significant. The program also reduced subjective fatigue and injuries during the annual training cycle, with fewer injuries and lower post-training fatigue in the EXP group. A lower number of injuries was associated with higher flexibility levels, suggesting that increased flexibility may reduce injury risk. Additionally, a strong positive correlation was found between RPE and injury incidence. The results indicate that traditional high-intensity warm-ups may not provide optimal preparation from a health-oriented perspective. This study highlights the importance of innovative training interventions and supports the multidimensional development of young athletes’ health profiles. Future studies should explore similar interventions in other team sports disciplines.

### Practical Implications

The obtained results generate important practical applications for coaches and young football players in this flagship team sport discipline. The implemented experimental programme can be considered an effective, practice-oriented intervention aimed at shaping and enhancing the sport-specific and health-oriented profile of young athletes. This protocol can be integrated into training cycles—particularly during the preparatory period, when optimal readiness for the competitive phase is essential—as a complementary functional method for warm-up and cooldown. Combined with regular high-intensity main-part training, it may optimise (1) health-related injury prevention and (2) training and competitive effectiveness, emphasising the practical value of the rational strategy within the broader training process. The protocol can be safely and easily implemented in standard training conditions. It is also advisable to adapt the intervention framework to other team sport disciplines.

## Figures and Tables

**Table 1:** Statistical characteristics of age, basic somatic traits, and training experience of the studied junior football players (n = 60): EXP (n = 30) vs. CON (n = 30).

Variable	$\mathrm{Group}\;\mathrm{EXP}\;(n\;=\;30);\;\tilde{x}$ ± sd	$\mathrm{Group}\;\mathrm{CON}\;(n\;=\;30);\;\tilde{x}$ ± sd	*p*
Body height [cm]	172.23 ± 6.71	171.92 ± 6.96	0.842
Body mass [kg]	70.46 ± 6.15	70.15 ± 6.26	0.873
BMI [kg/m^2^]	23.64 ± 1.82	23.60 ± 1.86	0.918
Age [years]	16.55 ± 0.50	16.56 ± 0.52	0.974
Training experience [years]	7.60 ± 1.20	7.54 ± 1.25	0.889

$\tilde{x}$—arithmetic mean; sd—standard deviation; *p*—level of significance.

**Table 2:** Statistical characteristics and comparative analyses (between-group and within-group) of flexibility and motor performance variables in the examined youth football players.

Measurement	Group EXP (n = 30)			Group CON (n = 30)			d_1_	*p*_1_	*d_c_*_1_
	$\tilde{x}$	sd	CV%	$\tilde{x}$	sd	CV%			
**Forward Trunk Flexion in Standing Position [cm]**									
I pre-test	10.43	1.05	10.04	10.66	0.99	9.24	−0.23	0.388	0.23
II post-test	11.56	1.10	9.55	10.78	0.81	7.53	0.78	0.003 *	0.82 ^S^
d_2_	1.13			0.12			-	-	-
*p*_2_	<0.001 **			0.642			-	-	-
*d_c_*_2_	1.05 ^S^			0.13			-	-	-
**Standing Long Jump [cm]**									
I pre-test	218.44	7.91	3.62	219.59	8.11	3.69	−1.15	0.584	0.14
II post-test	219.71	7.74	3.52	219.63	8.02	3.65	0.08	0.970	0.01
d_2_	1.27			0.04			-	-	-
*p*_2_	0.278			0.846			-	-	-
*dc*_2_	0.16			0.00			-	-	-
**10 × 5 m Shuttle Run [s]**									
I pre-test	19.92	1.08	5.42	19.61	1.33	6.78	0.31	0.487	0.26
II post-test	19.52	1.04	5.33	19.59	1.40	7.15	−036	0.798	0.06
d_2_	−0.4			−0.02			-	-	-
*p*_2_	0.152			0.912			-	-	-
*dc*_2_	0.38 ^M^			0.01			-	-	-
**Flamingo Balance [s]**									
I pre-test	6.50	5.12	78.77	6.11	5.44	89.04	0.39	0.719	0.07
II post-test	7.15	5.01	70.06	6.38	5.38	84.32	0.77	0.518	0.15
d_2_	0.65			0.27			-	-	-
*p*_2_	0.326			0.451			-	-	-
*dc*_2_	0.13			0.05			-	-	-

$\tilde{x}$—arithmetic mean; sd—standard deviation; CV%—coefficient of variation; I—first measurement period (pre-test); II—second measurement period (post-test); d—difference between means (delta): _1_—differences in intergroup means; _2_—differences in intragroup means; *p*—level of significance: _1_—intergroup; _2_—intragroup; * statistically significant values (*p* < 0.05); ** statistically significant values (*p* < 0.001); *d_C_*_1_—effect size expressed using Cohen’s *d* coefficient (intergroup); *d_C_*_2_—effect size expressed using Cohen’s *d* coefficient (intra-group); ^M^ moderate effect; ^S^ strong effect.

**Table 3:** Statistical Characteristics and Between-Group Comparative Analysis of Subjective Exertion (RPE) and Retrospective Injury Incidence in the Annual Training Cycle among Youth Football Players.

Variables	Group EXP (n = 30)			Group CON (n = 30)			d	*p*	*d_c_*
	$\tilde{x}$	sd	CV%	$\tilde{x}$	sd	CV%			
Subjective Rating of Perceived Exertion (RPE) [points]	6.86	0.82	11.32	7.22	0.73	9.41	−0.36	0.016 *	0.46 ^M^
Lower-Limb and Trunk Injuries in the Annual Training Cycle [n]	0.97	0.83	83.84	1.33	0.66	47.20	−0.36	0.026 *	0.48 ^M^

$\tilde{x}$—arithmetic mean; sd—standard deviation; CV%—coefficient of variation; d—difference between means (delta); *p*—level of significance; * statistically significant values (*p* < 0.05); *d_C_*—effect size expressed using Cohen’s *d* coefficient; ^M^ moderate effect.

**Table 4:** Correlation coefficients for the examined measurement variables in youth football players.

Variable Pairs	Group EXP (n = 30)		Grupa CON (n = 30)	
	r_p_	*p*	r_p_	*p*
Flexibility vs. Lower-limb injuries	−0.52	0.007 *	−0.56	0.004 *
Subjective effort rating (RPE) vs. Lower-limb injuries	0.72	<0.001 **	0.61	0.002 *
Training experience vs. Lower-limb injuries	−0.53	0.012 *	−0.68	0.001 *
Training experience vs. Subjective effort rating (RPE)	−0.50	0.018 *	−0.70	<0.001 **

r_p_—value of the correlation coefficient Pearson; *p*—level of significance; * a statistically significant level of differentiation (*p* < 0.05); ** statistically significant values (*p* < 0.001).

## Data Availability

The data presented in this study are available upon request from the corresponding author.
